# Effect of gonadectomy and estradiol on the expression of insulin signaling cascade genes in female and male mice

**DOI:** 10.18699/VJ20.635

**Published:** 2020-07

**Authors:** T.V. Iakovleva, N.E. Kostina, E.N. Makarova, N.M. Bazhan

**Affiliations:** Institute of Cytology and Genetics of Siberian Branch of the Russian Academy of Sciences, Novosibirsk, Russia; Institute of Cytology and Genetics of Siberian Branch of the Russian Academy of Sciences, Novosibirsk, Russia; Institute of Cytology and Genetics of Siberian Branch of the Russian Academy of Sciences, Novosibirsk, Russia; Institute of Cytology and Genetics of Siberian Branch of the Russian Academy of Sciences, Novosibirsk, Russia Novosibirsk State University, Novosibirsk, Russia

**Keywords:** gonadectomy, estradiol, testosterone, insulin sensitivity, gene expression, C57BL/6J mice, гонадэктомия, эстрадиол, тестостерон, чувствительность к инсулину, экспрессия генов, мыши линии C57BL/6J

## Abstract

A positive effect of estradiol on insulin sensitivity has been shown for females and males. Insulin sensitivity is higher in females than in males, and males show a greater tendency to develop metabolic disorders. It is believed that these sex differences are due to a protective effect of estradiol in females, but not in males. Estradiol is a steroid hormone, and its effect is due to the modulation of target gene expression, but the effect of estradiol on the expression of genes encoding insulin signal transduction and glucose transport has not been sufficiently studied. The aim of the study was to compare the molecular mechanisms of the estradiol influence on insulin sensitivity in mice of both sexes. The effect of gonadectomy and estradiol (1 μg/animal, three days) on the expression of insulin signaling cascade genes in muscle, adipose tissue, and liver, as well as on the expression of Fgf21, estradiol receptors (Esr1/2), and transcription factor Stat3 in the liver in female and male mice was investigated. Estradiol levels were lower and glucose blood levels and insulin resistance were higher in Sham operated (Sham) males compared to Sham females. Irs2, Pik3cd, and Esr1/2 mRNA levels were lower in the liver of Sham males than in Sham females. In females, gonadectomy reduced the level of estradiol in the blood, increased insulin resistance and blood glucose levels compared to Sham females. Administration of estradiol to gonadectomized females decreased blood insulin levels and insulin resistance. In males, gonadectomy, on the contrary, increased the blood estradiol level, decreased blood insulin level and insulin resistance. Estradiol did not affect the parameters studied in males. The development of insulin resistance in gonadectomized females was associated with a decreased expression of the Irs2 gene in the liver. Increased insulin sensitivity in gonadectomized males was associated with increased levels of Irs2 and Pik3cd mRNA in the liver. It can be assumed that increasing the level of estradiol in the blood activates the expression of the Irs2 gene in the liver regardless of animal sex. Also, estradiol seems to regulate the transport of glucose in adipose tissue regardless of animal sex: in females and males, an increase in the blood estradiol level was associated with a decrease in the expression of the Slc2a4 gene in adipose tissue. Thus, the effects of estradiol on the expression of insulin cascade genes do not seem to depend on animal sex, but have tissue specificity. Since the molecular mechanism of estradiol influence on the expression of insulin cascade genes in females and males is the same, the cause of sexual differences in insulin sensitivity and the rate of development of metabolic disorders may be a decrease in the level of estradiol in the blood, as well as a decrease in the expression of estradiol receptors in the liver in males compared to females.

## Abstract (ru)

В настоящее время показано положительное влияние эстрадиола на чувствительность к инсулину на уровне целого организма у самок и самцов мышей. При этом чувствительность к инсулину в целом у са- мок выше, чем у самцов, и самцы демонстрируют большую склонность к развитию метаболических нарушений. Предполагают, что данные половые различия объясняются протективным действием эстрадиола у самок, но не у самцов. Эстрадиол является стероидным гормоном, и его действие обусловлено модуляцией экспрессии генов-мишеней, однако влияние эстрадиола на экспрессию генов, кодирующих трансдукцию сигнала инсулина и транспорт глюкозы в клетку, изучено недостаточно. Целью работы было сравнительное исследование моле- кулярных механизмов влияния эстрадиола на чувствительность к инсулину у мышей обоих полов. Исследовано влияние гонадэктомии и эстрадиола (1 мкг/животное, три дня) на экспрессию генов сигнального каскада инсу- лина в мышцах, жировой ткани и печени, а также на экспрессию Fgf21, рецепторов эстрадиола (Esr1/2) и транс- крипционного фактора Stat3 в печени у самок и самцов мышей. Ложно оперированные (ЛО) самцы отличались от ЛО самок сниженным уровнем эстрадиола, повышенным уровнем глюкозы и большей резистентностью к инсулину. В печени у ЛО самцов уровни мРНК Irs2, Pik3cd и Esr1/2 были ниже, чем у ЛО самок. У самок гонадэк- томия снижала уровень эстрадиола в крови, повышала резистентность к инсулину и уровень глюкозы в крови по сравнению с ЛО самками. Введение эстрадиола гонадэктомированным самкам снижало уровень инсулина в крови и резистентность к инсулину. У самцов гонадэктомия, наоборот, повышала уровень эстрадиола в крови, снижала резистентность к инсулину и уровень инсулина в крови. Введение эстрадиола гонадэктомированным самцам не оказывало влияния на исследованные показатели. Развитие инсулинорезистентности у гонадэкто- мированных самок было ассоциировано со снижением экспрессии гена Irs2 в печени, а повышение чувстви- тельности к инсулину у гонадэктомированных самцов – с увеличением уровней мРНК Irs2 и Pik3cd в печени. Можно предположить, что повышение уровня эстрадиола в крови активирует экспрессию гена Irs2 в печени не- зависимо от пола животного. Также независимо от пола животного эстрадиол, по-видимому, регулирует транс- порт глюкозы в жировой ткани: у самок и самцов повышение уровня эстрадиола в крови было ассоциировано со снижением экспрессии гена Slc2a4 в жировой ткани. Таким образом, эффекты эстрадиола на экспрессию ге- нов инсулинового каскада, по-видимому, не зависят от пола животного, но имеют тканевую специфичность. Поскольку молекулярный механизм влияния эстрадиола на экспрессию генов инсулинового каскада у самок и самцов не различается, причиной половых различий в чувствительности к инсулину и скорости развития метаболических нарушений может быть сниженный, по сравнению с самками, уровень эстрадиола в крови и сниженная экспрессия рецепторов эстрадиола в печени.

## Introduction

Current data suggest that there is a close relationship between estrogens and insulin sensitivity: estradiol increases the uptake of glucose in muscle, suppresses hepatic glucose production, lowers blood glucose levels and increases glucose tolerance in ovariectomized females of mice and rats, in intact female mice with severe genetic or diet-induced obesity, in male mice and in men (Faustini-Fustini et al., 1999; Bryzgalova et al., 2008; Saengsirisuwan et al., 2009; Zhu et al., 2014).

Molecular mechanisms of the estradiol effect on insulin sensitivity are being actively studied. It has already been shown that they are due to its effect on the phosphorylation of insulin receptor substrates (IRS1 and 2), as well as its effect on the glucose transporter 4 (GLUT4) level and GLUT4 translocation into the cell membrane (González et al., 2001; Saengsirisuwan et al., 2009; Gorres et al., 2011; Muthusamy et al., 2011; Narasimhan et al., 2013).

The effects of estradiol as a steroid hormone are related to its effect on gene expression. Currently, the effect of estradiol on the expression of the glucose transporter 4 gene (Slc2a4 ) in females and males and on the expression of the insulin receptor gene (Insr) in males has been studied. Ovariectomy has been shown to increase Slc2a4 expression in adipose tissue in female mice and estradiol administration to reduce it (Iakovleva et al., 2014). Gonadectomy reduces the expression of Insr in the liver, muscle and adipose tissue, and reduces the expression of Slc2a4 in muscle and adipose tissue, but exogenous estradiol does not affect the expressions in male rats (Muthusamy et al., 2009, 2011). The results of in vitro experiments performed on cell cultures (CHO, HepG2) suggest that estradiol does not participate in the regulation of Insr expression and activates the gene expression of the insulin receptor substrate 1 and 2 (Irs1/2) in the liver (Xie et al., 2003; Panno et al., 2006; Parthasarathy et al., 2009).

The estradiol effect on the expression of insulin cascade genes may be mediated by other factors. For example, the effect of estradiol on insulin sensitivity in mice with genetic obesity (ob/ob mice ) is due to activation of liver expression of the transcription factor STAT3 (Gao et al., 2006). Fibroblast growth factor 21 (FGF21) increases liver insulin sensitivity (Gong et al., 2016) and may also mediate the effect of estradiol on metabolism, because activation of the estradiol receptor alpha increases the liver expression of Fg f 21 in female mice (Allard et al., 2019).

Estrogens are known to be synthesized in the ovaries, testicles and adrenal glands, as well as in peripheral tissues from androgen precursors under the influence of an aromatase enzyme complex, so the blood estradiol level of male mice is comparable to that of females. However, males show a greater tendency to develop metabolic disorders and reduced insulin sensitivity when consuming high-fat food. In mice, high-fat diet reduces hepatic insulin sensitivity and induces fasting hyperglycemia in males, unlike females (Akoum et al., 2011). It is assumed that gender-related differences in insulin sensitivity and in the development rate of metabolic disorders are due to the fact that in females, unlike males, estradiol has a protective effect and increases insulin sensitivity. However, the molecular mechanisms of the estradiol effect on insulin sensitivity in males remain poorly understood.

The aim of the work was to perform a comparative study of the molecular mechanisms of the estradiol influence on insulin sensitivity in mice of both sexes. The effects of gonadectomy and exogenous estradiol on the expression of insulin signaling cascade genes in muscle, adipose tissue, and liver, as well as on the liver expression of Fg f 21, estradiol receptors of type alpha and beta (Esr1/2), and transcription factor Stat3 in female and male mice were studied.

## Materials and methods

**Animals.** C57BL/6J mice were kept in the vivarium of the Institute of Cytology and Genetics. The mice were housed under a 12:12-h light-dark regime at an ambient temperature of 22 °C. The mice were provided ad libitum access to commercial mouse chow (Assortiment Agro, Turakovo Village, Moscow oblast, Russia) and water. All experiments were performed according to the European Convention for the Protection of Vertebrate Animals used for Experimental and other Scientific Purposes (Council of Europe No. 123, Strasbourg 1985) and Russian national instructions for the care and use of laboratory animals. The protocols were approved by the Independent Ethics Committee of the Institute of Cytology and Genetics, Siberian Branch of the Russian Academy of Sciences.

**Experiment.** At the age of 10 weeks, females and males were gonadectomized and housed individually. Three weeks after the operation, three experimental groups were formed for each sex: sham surgery animals that received oral administration of oil (SHAM) and served as controls, gonadectomized animals that received an oil administration (GE) or 17β-estradiol administration (Е2). Animals received an oral administration of β-estradiol (Sigma-Aldrich) at a dose of 1 μg/animal or a solvent (vegetable oil, 100 μl) for three days at 09:00. A day after the last administration, the animals were decapitated after a night of fasting (18:00–09:00). Blood and tissue samples (liver, muscle, visceral fat) were collected. Blood was collected in tubes with 5 μl of EDTA and centrifuged (4000 g, 20 minutes), and blood plasma was stored at –70 °C. Tissue samples were stored in liquid nitrogen until RNA and protein were isolated. After determining the fasting plasma levels of glucose and insulin, the physiological index of insulin resistance (HOMA-IR) was calculated using the formula [plasma glucose level (mmol/l) × plasma insulin level (ng/ml)]/22.5.

**The reaction of reverse transcription and real-time PCR.** The total RNA was isolated using the ExtraRNA reagent (Eurogen Lab, Moscow, Russia) in accordance with the manufacturer’s instructions. First-strand cDNA was synthesized with Moloney murine leukemia virus (MMLV) reverse transcriptase buffer (SibEnzyme, Novosibirsk, Russia) and oligo (dT) (Evrogen, Moscow, Russia) as a primer. Applied Biosystems TaqMan gene expression assays (Table 1) with β-actin as endogenous control and 2.5× reaction mixture for qPCR in the presence of Rox reference dye (Syntol, Moscow, Russia). Real-time PCR was performed using Applied Biosystems ViiA™ 7 Real-Time PCR System, using the standard protocol according to the manufacturer’s instructions (Applied Biosystems). Relative quantitation was performed by the comparative CT method, where CT is the threshold cycle.

**Table 1.**
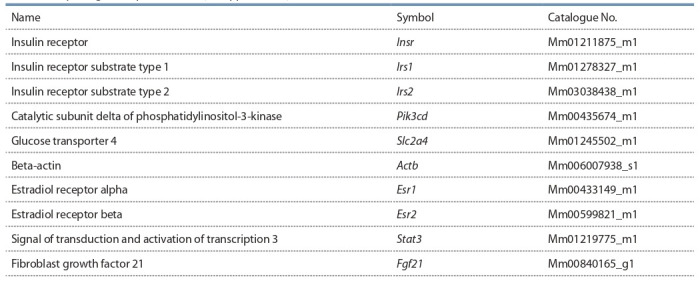
Taqman gene expression assays (Applied Biosystems ) for mice used in the work

**Western blot analysis of protein levels.** Samples of liver, muscle and adipose tissue were homogenized. Protein extraction was performed in a lysing buffer (Tris-Triton buffer). The protein concentration in the samples was evaluated using the Bradford method using NanoDrop2000 (ThermoScientific). Protein separation by molecular weight was performed using gel electrophoresis in 10 % polyacrylamide gel in Tris-glycine buffer (25 mm Tris, 250 mm Glycine, 0.1 % SDS). Electric transfer of proteins to a 0.45 micron nitrocellulose membrane was performed using the Trans-Blot system (Bio-Rad, USA). The membranes were blocked with 5 % milk (milk powder, PanReac AppliChem). Primary polyclonal rabbit antibodies were used (Santa Cruz Biotechnology, USA, breeding 1:2000): insulin Rα antibody (sc-710) and GLUT4 antibody (sc-7938). After washing with a phosphate-salt buffer (0.1 % Tween-20), the membranes were incubated for 1 hour at room temperature with secondary goat antibodies conjugated with horseradish peroxidase (1:5000 dilution) (sc-2004, At/goat anti-rabbit IgG-HRP, HRP-conjugated, Santa Cruz Biotechnology, USA). Detection of the structural protein beta-actin (1:5000 dilution) (sc-130656, Santa Cruz Biotechnology, USA) was performed on the same membrane. At the end of immunoblotting, the membrane was washed and incubated for 1 minute in a substrate mixture (10 ml 100 mm Tris-Hcl pH 8.5; 50 μl 250 mM luminol; 22 μl 90 mM coumaric acid; 3 μl 33 % H2O2), after which chemiluminescence was visualized on the ChemiDocTM XRS device (Bio-Rad, USA). The results were analyzed using the Image Studio Lite Ver 5.2 program. The signal of the test protein in the sample was related to the beta actin signal in the same sample. The level of protein expression in a sample is the ratio of the normalized signal in this sample to the normalized signal in the reference sample.

**Determination of blood biochemical parameters.** Blood glucose concentration was determined using the OneTouch Select glucometer (Lifescan, Johnson and Johnson, USA). Concentrations of estradiol, testosterone, and insulin in blood plasma were determined by the ELISA method using commercial kits (Mouse Estradiol (E2) ELISA Kit (MyBioSource, USA), Testosterone rat/mouse ELISA (Demeditec Diagnostics GmbH, Germany) and Rat/Mouse Insulin ELISA Kit (Millipore, USA)) according to manufacturers’ instructions.

Statistical analysis. The results are presented as means ± SE from the indicated number of mice. The effect of gonadectomy and exogenous estradiol on the studied parameters in females and males was determined using a single-factor MANOVA variance analysis (gradations of the factor “experimental group”: SHAM, GE, E2) with multiple comparisons using the post hoc Newman–Keuls test. MANOVA with gradations of the factor “experimental group” SHAM and GE was used to analyze the effect of gonadectomy on the plasma estradiol level, since blood samples were taken a day after the last injection of the hormone, and the level of estradiol in the blood of animals E2 could not reflect the actual level of the hormone in the blood after injection. To compare the parameters of SHAM females and SHAM males, a t-test was used. Significance was determined as p < 0.05.

## Results

**Blood levels of sex hormones, glucose, and insulin**

**Sex effects (SHAM mice).** In females, the estradiol level was significantly higher, and the testosterone level was significantly lower than in males (Table 2). Females had a higher sensitivity to insulin than males: the insulin level of females and males did not differ significantly, while the glucose level and the index of insulin resistance (HOMA-IR) in females was significantly lower than in males (Table 3).

**Table 2.**
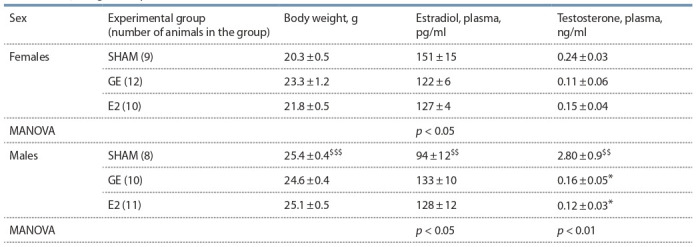
Body weight and plasma levels of sex hormones in female and male C57BL mice $$ p < 0.01, $$$ p < 0.001 compared to SHAM females, t-test; * p < 0.05 compared to SHAM animals of the same sex, post hoc Newman–Keuls test.

**Table 3.**
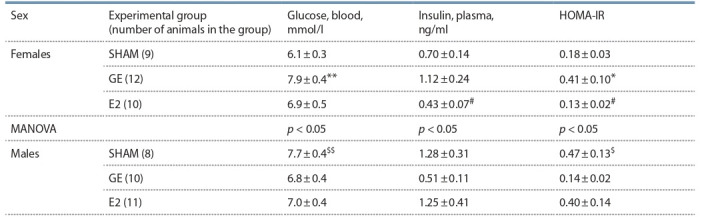
Plasma insulin levels, blood glucose levels, and HOMA-IR in C57BL females and males $ p < 0.05, $$ p < 0.01 compared to SHAM females, t-test; * p < 0.05, ** p < 0.01 compared to SHAM animals of the same sex; # p < 0.05 compared to GE animals of the same sex, post hoc Newman–Keuls test.

**Effect of gonadectomy and exogenous estradiol.** In females, gonadectomy reduced the plasma estradiol level (MANOVA, p <0.05). A significant influence of the “experimental group” on the insulin sensitivity in females was shown: the index of insulin resistance, glucose and insulin levels in GE females were higher than in SHAM females, and exogenous estradiol normalized these indicators.

In males, gonadectomy reduced the plasma testosterone level (MANOVA, p < 0.01). On the contrary, the plasma estradiol level in GE males was higher than that of SHAM males (MANOVA, p <0.05). Gonadectomy and estradiol did not affect blood glucose and insulin levels and the index of insulin resistance in males.

**Expression of insulin cascade components in the liver**

**Fig. 1.**
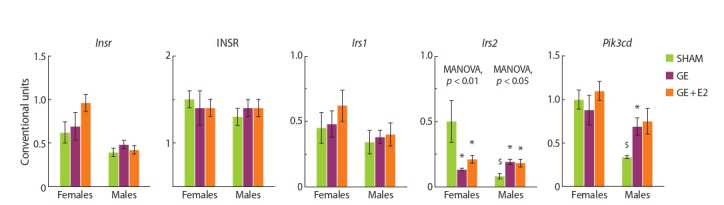
Effect of gonadectomy and exogenous estradiol (1 μg/animal, 3 days) on mRNA levels of Insr, Irs1, Irs2, Pik3cd and the level of INSR protein in the liver in SHAM, GE and GE + E2 female and male mice. Here and in the Fig. 2–4: $ p < 0.05 compared to females; * p < 0.05 compared to SHAM animals of the same sex; # compared to GE animals of the same sex. MANOVA, p < 0.05 or p < 0.01 – the influence of the “experimental group” factor is statistically significant.

**Fig. 2.**
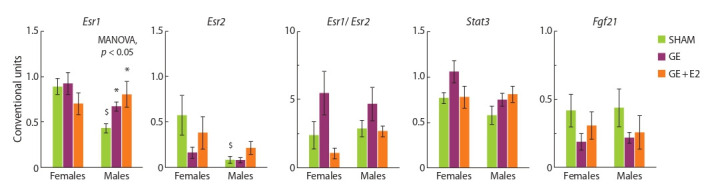
Effect of gonadectomy and exogenous estradiol (1 μg/animal, 3 days) on the mRNA levels of estradiol receptors (Esr1 and Esr2), Fgf21 and Stat3 in the liver in SHAM, GE and GE + E2 female and male mice.

**Sex effects (SHAM mice).** The expression of Irs2, Pik3cd, Esr1, and Esr2 in females differed from that in males: the mRNA level of these genes was significantly higher in females than in males. The expression of Insr, Irs1, Fg f 21, and Stat3 and the level of the INSR protein were not significantly different in females and males (Fig. 1 and 2).

**Effect of gonadectomy and exogenous estradiol.** In females, gonadectomy decreased, while exogenous estradiol increased, although not normalized, the Irs2 mRNA level in the liver (MANOVA, p < 0.01).

the studied genes, while gonadectomy increased hepatic expression of Irs2 and Esr1 (MANOVA, p < 0.05 in both cases): the mRNA levels of these genes in GE and E2 males were higher than in SHAM males. The level of Pik3cd mRNA in the liver of GE and E2 males was also higher than in SHAM males, but the differences did not reach the level of significance (MANOVA, p = 0.07). The level of Esr1 mRNA in males was positively correlated ( p < 0.05) with the level of Irs2 mRNA (r = 0.74).

Gonadectomy and estradiol did not significantly affect the Esr1/Esr2 ratio in the liver in females and males.

**Expression of components of the insulin cascade in muscle and adipose tissues**

**Fig. 3.**
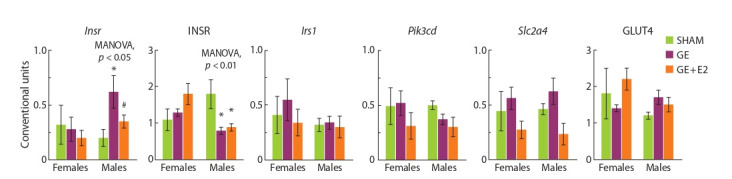
Effect of gonadectomy and exogenous estradiol (1 μg/animal, 3 days) on the mRNA levels of Insr, Irs1, Pik3cd, Slc2a4 and the level of INSR and GLUT4 proteins in skeletal muscles in SHAM, GE and GE + E2 female and male mice.

**Fig. 4.**
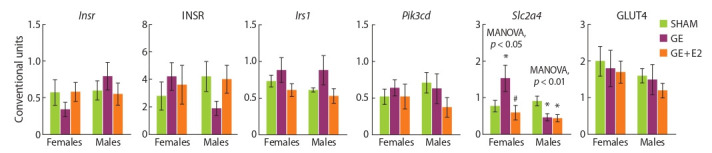
Effect of gonadectomy and exogenous estradiol (1 μg/animal, 3 days) on the mRNA levels of Insr, Irs1, Pik3cd, Slc2a4 and the level of INSR and GLUT4 proteins in visceral adipose tissue in SHAM, GE and GE + E2 female and male mice.

**Sex effects (SHAM mice).** The expression of insulin cascade genes and proteins in muscle and adipose tissue did not differ in SHAM females and SHAM males (Fig. 3 and 4).

Effect of gonadectomy and exogenous estradiol. In females, gonadectomy and estradiol did not affect the expression of the studied parameters of the insulin cascade in muscle tissue. In GE males, the Insr mRNA level in muscle was higher than in SHAM males, and the INSR protein level was lower in GE and E2 males, compared to SHAM males. In females, gonadectomy increased and exogenous estradiol normalized Slc2a4 expression in adipose tissue (MANOVA, p < 0.05). In GE and E2 males, Slc2a4 expression in adipose tissue was lower than in SHAM males.

## Discussion

One approach to study the effect of estradiol on the expression of genes involved in insulin signal transduction is comparison of females and males. Insulin sensitivity at the whole body level (blood glucose level, insulin resistance index), as well as hepatic expression of insulin signal transduction genes (Irs2 and Pik3cd ) in SHAM females was higher than that in SHAM males, which coincides with data obtained on intact animals (Parks et al., 2015; Torre et al., 2017; Yakovleva et al., 2017). It was shown for the first time that SHAM females differed from SHAM males not only in increased blood estradiol levels, but also in increased expression of both types of estradiol receptors in the liver, which can be one of the causes of sex differences in effects of estradiol on insulin sensitivity in the liver.

To study the effects of estradiol on the expression of insulin cascade genes, in addition to comparing parameters in females and males, we used a model of gonadectomy with subsequent administration of estradiol. We assumed that gonadectomy would lead to a decrease in the blood estradiol level in females as a result of elimination of the main source of hormone production, and in males – as a result of a decrease in the level of testosterone, as a precursor of estradiol synthesis. However, in males, the level of the hormone in the blood after gonadectomy increased. This result may be due to the activation of hormone production by the adrenal glands. As a result, gonadectomy eliminated differences in the level of sex steroids between females and males: the blood estradiol and testosterone levels did not differ in the gonadectomized females and males. However, in females, gonadectomy induced the development of insulin resistance, and exogenous estradiol normalized insulin sensitivity, while in males, gonadectomy and estradiol did not have a significant effect on the studied parameters of insulin sensitivity (blood glucose and insulin levels, HOMA-IR). The results of the effect of ovariectomy and exogenous estradiol on blood glucose and insulin levels and the index of insulin resistance in females correspond to existing data (Rogers et al., 2009; Oh et al., 2011). Effects of gonadectomy on HOMA- IR in males, as shown by Parks and co-authors (Parks et al., 2015), depends on the animal’s genotype, and in C57BL/6J males, it decreases 10 weeks after gonadectomy. In this study, the HOMA-IR index in GE males did not differ significantly, but was 3.4 times lower than in SHAM males. The absence of a significant effect of gonadectomy on insulin sensitivity in males may be due to the shorter duration of the experiment.

In GE females, decreased insulin sensitivity was associated with decreased hepatic expression of Irs2 and Esr2 and increased expression of Slc2a4 in adipose tissue. Exogenous estradiol, in contrast, reduced Slc2a4 expression in adipose tissue and increased Irs2 expression in the liver. The effect of gonadectomy on hepatic Irs2 expression is well consistent with the observed sexual differences: Irs2 expression in females was higher than in males. The effects of estradiol on the hepatic expression of Irs1 and Irs2 in female mice are known to mediate estradiol type alpha (ERα) receptors (Panno et al., 2006). Estradiol beta-type receptors (ERβ) are thought to inhibit the estradiol effects mediated by ERα (Lindberg et al., 2003). According to the obtained data, ovariectomy did not affect ERα expression and reduced ERβ expression in the liver in females, which implies an increase in the estradiol effects mediated by ERα, and may have a compensatory-adaptive effects to maintain insulin sensitivity in conditions of reduced blood estradiol levels.

In males, gonadectomy did not affect blood insulin and glucose levels, but caused increased levels of Irs2, Pik3cd, and Esr1 mRNA in the liver. Since males have a tendency to increase the level of estradiol in the blood after gonadectomy, and there is a correlation between the level of Irs2 expression and Esr1 expression in the liver, it can be assumed that the hepatic Irs2 expression in males, as in females, is regulated by estradiol. Accordingly, activation of the gene expression of the estradiol receptor alpha may be part of the molecular mechanism of the estradiol effect on insulin sensitivity in GE males. Thus, increasing the blood estradiol level in females and males can activate the Irs2 gene expression in the liver regardless of gender and contribute to improving insulin sensitivity in general.

Transcription factor STAT3 was shown to mediate the estradiol effects on the expression of hepatic lipogenic genes (Gao et al., 2006) and FGF21 may mediate the estradiol effect on the expression of gluconeogenic genes, since it increases the gene expression of Irs2 and the glucose-6-phosphatase (Fisher et al., 2011). However, the role of STAT3 and FGF21 in mediating the estradiol effects on the expression of insulin signal transduction genes requires additional research, since in the study no differences were found in the Stat3 and Fg f 21 mRNA levels in the liver in animals of different sexes and experimental groups.

Activation of liver Pik3cd expression in GE males appears to be due to a decrease testosterone levels. In females, the Pik3cd mRNA level was higher than in males, but these differences were not related to estradiol levels, since ovariectomy and subsequent estradiol administration did not affect the level of Pik3cd mRNA in females.

It is believed that the effect of estradiol on insulin sensitivity in adipose and muscle tissues is due to its stimulation of glucose uptake by cells as a result of increasing the level of GLUT4 and activating its translocation into the cell membrane. In female mice, ovariectomy was shown to have no effect after 2 weeks, and caused a decrease in the level of Slc2a4 mRNA in muscle and adipose tissue after 10 weeks (Kim et al., 2010). In female rats, 12 weeks after ovariectomy, the level of GLUT4 protein in the muscles is reduced, while the estradiol injections prevents this decrease (Saengsirisuwan et al., 2009). In this study, 3 weeks after ovariectomy, the level of Slc2a4 mRNA in adipose tissue in females increased and exogenous estradiol normalized it, while there was no significant effect on the level of Slc2a4 mRNA in muscle tissue and the level of GLUT4 protein in adipose tissue and muscle. We have previously shown that ovariectomy for 5 weeks also increases the level of Slc2a4 mRNA in adipose tissue, and estradiol for 3 weeks reduces it, while in muscle tissue the level of Slc2a4 mRNA decreases after ovariectomy, but exogenous estradiol does not affect it (Iakovleva et al., 2014). Apparently, the effect of ovariectomy and exogenous estradiol on Slc2a4 expression in adipose and muscle tissues depends significantly on the duration of the experiment.

The effect of gonadectomy and estradiol on the expression of insulin receptor and glucose transporter 4 in adipose and muscle tissues was studied in male rats. Gonadectomy has been shown to decrease levels of mRNA and protein of INSR and protein level of GLUT4 in adipose and muscle tissues, and exogenous estradiol normalizes levels of these proteins (Muthusamy et al., 2009, 2011). The results of our experiment, obtained on mice, are poorly consistent with these data. This may be due to interspecies differences in the influence of gonadectomy on the blood estradiol levels and sex steroid ratio in males. In our experiment, an increase in estradiol levels after gonadectomy in males was associated with an increase in Insr mRNA, but a decrease in INSR protein in muscle, and a decrease in Slc2a4 mRNA in adipose tissue. It should be noted that an increase in the blood estradiol levels (as a result hormone administration in females and after gonadectomy in males) was associated with a decrease in the Slc2a4 expression in adipose tissue regardless of sex.

## Conclusion

All of the above suggests that the effect of estradiol on the expression of genes and proteins of the insulin cascade is tissue-specific and does not depend on the sex: estradiol can increase the expression of Irs2 in the liver, and can suppress the expression of Slc2a4 in adipose tissue. Activation of Irs2 expression in the liver when the blood estradiol level increases causes an improvement in glucose metabolism, so the effects of estradiol in the liver cause an increase in insulin sensitivity at the whole body. The significance of the estradiol effect on Slc2a4 expression in adipose tissue in females and males is not clear and requires further research. Despite the universal mechanism of action, the protective effect of estradiol in males is less pronounced than in females, apparently as a result of reduced hormone levels in blood and reduced expression of estradiol receptors in the liver.

## Conflict of interest

The authors declare no conflict of interest.
